# Maternal Enterovirus Infection during Pregnancy as a Risk Factor in Offspring Diagnosed with Type 1 Diabetes between 15 and 30 Years of Age

**DOI:** 10.1155/2008/271958

**Published:** 2008-07-28

**Authors:** Maria Elfving, Johan Svensson, Sami Oikarinen, Björn Jonsson, Per Olofsson, Göran Sundkvist, Bengt Lindberg, Åke Lernmark, Heikki Hyöty, Sten-Anders Ivarsson

**Affiliations:** ^1^Department of Clinical Sciences, Pediatric Unit, Lund University Hospital, Lund University, 221 85 Lund, Sweden; ^2^Department of Clinical Sciences, Pediatric Unit, Malmö University Hospital, Lund University, 205 02 Malmö, Sweden; ^3^Department of Virology, Tampere University Hospital, University of Tampere, 33521 Tampere, Finland; ^4^Department of Women's and Children's Health, Uppsala University, 751 05 Uppsala, Sweden; ^5^Department of Clinical Sciences, Obstetrics and Gynecology Unit, Malmö University Hospital, Lund University, 205 02 Malmö, Sweden; ^6^Department of Clinical Sciences, Diabetes and Celiac Disease Unit, Malmö University Hospital, Lund University, 205 02 Malmö, Sweden; ^7^Department of Medicine, University of Washington, Seattle, WA 98195, USA

## Abstract

Maternal enterovirus infections during pregnancy may increase the risk of offspring developing type 1 diabetes during childhood. The aim of this study was to investigate whether gestational enterovirus infections increase the offspring's risk of type 1 diabetes later in life. Serum samples from 30 mothers without diabetes whose offspring developed type 1 diabetes between 15 and 25 years of age were analyzed for enterovirus-specific immunoglobulin M (IgM) antibodies and enterovirus genome (RNA), and compared to a control group. Among the index mothers, 9/30 (30%) were enterovirus IgM-positive, and none was positive for enterovirus RNA. In the control group, 14/90 (16%) were enterovirus IgM-positive, and 4/90 (4%) were positive for enterovirus RNA (n.s.). Boys of enterovirus IgM-positive mothers had approximately 5 times greater risk of developing diabetes (OR 4.63; 95% CI 1.22–17.6), as compared to boys of IgM-negative mothers (*P* < .025). These results suggest that gestational enterovirus infections may be related to the risk of offspring developing type 1 diabetes in adolescence and young adulthood.

## 1. INTRODUCTION

Type 1 diabetes develops in genetically susceptible individuals as a result of progressive autoimmune destruction of *β*-cells in the pancreas. The incidence of type 1 diabetes has increased worldwide in recent decades [1–4]. The peak age at onset is at 10 to 14 years of age, and while there is no difference in the incidence between boys and girls [4, 5], several studies have shown a gender difference after 15 years of age with a male:female ratio of approximately 3:2 [5–7]. The reason for this gender difference is not known but it cannot be excluded that susceptibility to environmental factors may contribute.

Several studies support the hypothesis that pre- and perinatal exposures to environmental risk factors are significant in the development of type 1 diabetes. Advanced maternal age at delivery, complications during delivery, delivery by cesarean section, and blood group incompatibility are related to increased risk of type 1 diabetes in childhood [8–12]. It is also thought that congenital rubella increases the risk of type 1 diabetes later in life, especially in the second and third decades [13–15]. Enterovirus infections are one of the main candidates for an environmental trigger of type 1 diabetes [16–20], and maternal enterovirus infections during pregnancy have also been associated with increased risk of offspring developing type 1 diabetes during childhood in the age group 0–14 years [21–24].

We have previously reported that cord blood islet autoantibodies did not affect the risk for type 1 diabetes in 15–30-year-old subjects [25]. Therefore, in this study, we examined whether intrauterine exposure to enterovirus infection in the 15–30-year-old ages was associated with an increased risk of offspring developing type 1 diabetes, also with particular reference to possible gender difference.

## 2. MATERIALS AND METHODS

### 2.1. Study population

The city of Malmö, Sweden, has 270 000 inhabitants who are served by Malmö University Hospital (U-MAS), its only hospital. The vast majority of deliveries in Malmö take place in the Department of Obstetrics at this hospital. Since 1970, umbilical cord blood serum has been taken at delivery from the majority of children born at Malmö University Hospital and stored, together with a maternal serum sample similarly taken at delivery, at –20°C. Among those children born from April 1970 to July 1984, 38 later developed type 1 diabetes between the ages of 15 and 30. They were identified using the Diabetes Incidence Study in Sweden (DISS) Registry and classification of type 1 diabetes in the offspring was done by the physicians, using clinical data and laboratory findings as detailed in the DISS-study [26]. Of the group, 32 had cord serum and a corresponding maternal serum sample saved from the time of birth. Two of the thirty-two mothers (6%) had type 1 diabetes and were excluded, since only 3% of new onset type 1 diabetes patients have a mother with the disease. Consequently, the study was comprised of 30 mothers whose offspring (14 males and 16 females) developed type 1 diabetes at a median age of 18 years (range 15.2–25.5). The median age of the mothers at delivery was 25.8 years (range 19.7–34.8). Information about the incidence of type 1 diabetes among the fathers was not available.

The control group consisted of 90 maternal serum samples, including three control mothers for each of the 30 case mothers, matched by date of delivery. The median age of the control group was 25.9 years (range 19.4–40.1). Three of the control mothers, although they were matched for month and day, gave birth in the following year. Altogether, there were 52 males and 38 females born to the control mothers. None of these children developed type 1 diabetes during the follow-up time.

### 2.2. Enterovirus antibodies

All sera were subjected to blind analysis. Immunoglobulin M (IgM) was measured using a capture enzyme immunoassay (EIA), as previously described [27]. A cocktail of heat-treated Coxsackievirus B3, Coxsackievirus A16, and echovirus 11 was used as the antigen in this assay, making it broadly reactive to different enterovirus serotypes. Biotinylated immunoglobulin G (IgG) class antibodies, purified from the serum of rabbits immunized with sucrose-gradient purified viruses (Coxsackievirus B3, Coxsackievirus A16, and echovirus 11), were used as detection antibodies in the laboratory. After washing, streptavidin-peroxidase conjugate 9534A (Bethesda Research Laboratories, Gaithersburg, Md, USA) was added. The level of IgM was expressed as positive (+) or negative (−). The cutoff limit for IgM positivity was three multiples of the background, which is the optic density (OD) value when the serum layer is replaced by phosphate-buffered saline [28]. Values exceeding the background by four times or more were considered highly positive.

### 2.3. Enterovirus RT-PCR

RNA was extracted according to the manufacturer's protocol by a QIAamp viral RNA kit (Qiagen, Hilden, Germany) from 140 *μ*L of the serum. Enterovirus RNA was detected by reverse transcription-polymerase chain reaction (RT-PCR) and a subsequent hybridization step that detects practically all enterovirus serotypes, as described elsewhere [29]. All positive samples were confirmed by repeated RT-PCR.

### 2.4. Statistical analysis

Nonparametric methods were applied. Group comparisons were performed using the Mann-Whitney test. Four field tables displaying the frequencies of study groups were analyzed by means of Fisher's exact test. Logistic regression analyses were also performed. A two-tailed *P* value <.05 was considered statistically significant. The statistical analyses were carried out with the standard statistical package (SPSS) for Windows, V15.0 (SPSS Inc, Chicago, Ill, USA).

## 3. RESULTS

The prevalence of enterovirus IgM in sera taken at delivery from mothers of children who developed type 1 diabetes and from control mothers is given in Table 1. There was no significant difference in ages between the groups of mothers. The prevalence value of enterovirus IgM was higher in the mothers whose offspring developed type 1 diabetes, as compared to control mothers, but the difference did not reach statistical significance (*P* < .11) (Table 1). Among the mothers of offspring with diabetes, 9/30 (30%) were enterovirus IgM-positive, and 5/30 (17%) had high IgM titers. None was positive for enterovirus RNA. In the control group, 14/90 (16%) were enterovirus IgM-positive, 8/90 (9%) had high titers, and 4/90 (4%) were positive for enterovirus RNA. No significant differences were found between the groups (Table 1).

The gender of the child did influence the risk of diabetes following maternal enterovirus infection (Table 2). In logistic regression controlling for mother's age and the interaction between IgM-positivity and gender we found that boys born to IgM-positive mothers showed an increased risk of developing type 1 diabetes (odds ratio [OR] 4.63; 95% confidence interval [CI] 1.22–17.6; *P* < .025), as compared to boys of IgM-negative mothers. No such increased risk was found in girls born to IgM-positive mothers (OR 0.21; 95% CI 0.03–1.56). Mother's age was included but was not a significant predictor of developing type 1 diabetes. The results were similar when logistic regression was done without controlling for maternal age. The frequency of maternal enterovirus IgM with regard to gender of the offspring is given in Table 3.

## 4. DISCUSSION

This study analyzed the correlation of maternal enterovirus infections during pregnancy and the future risk of type 1 diabetes occurring in the offspring. The presence of enterovirus antibodies (IgM) and enterovirus genome (RNA) was analyzed by means of stored serum samples obtained at delivery from 30 mothers without diabetes whose offspring subsequently developed type 1 diabetes during adolescence or young adulthood. Comparable samples were taken from 90 matched control mothers. The mothers of the offspring who later developed type 1 diabetes were carefully matched to control mothers and the two groups were expected to be exposed to a similar infectious environment during pregnancy. The strength of our study is that the countywide DISS registry [26] made it possible to identify not only the offsprings who developed diabetes but to ensure that none of the children of the control mothers had acquired the disease. The study is explorative due to the limited study cohort, but it is unique as the serum samples used were obtained from mothers who delivered their children as long as 30 years ago in the same hospital.

We observed a difference in the presence of enterovirus IgM between the patient and control groups, although it did not reach statistical significance. However, our study indicated that maternal enterovirus infection was a significant risk factor for the development of diabetes in boys, but not in girls. This finding suggests that boys may be more susceptible to the diabetogenic effect of enteroviruses than girls during the prenatal period. Prospective studies such as the ongoing DiPiS [30] and TEDDY [31] studies will be needed to fully establish if maternal enterovirus infections contribute to the gender difference in 15–25 year old type 1 diabetes patients.

Enterovirus RNA was only observed in a few control mothers. PCR of enterovirus is known to be positive in serum only for a period between a few days and 1-2 weeks during viremia. Therefore, a larger number of mothers who gave birth to children developing type 1 diabetes as 15–30 year olds would be needed to fully explore the possible role of gestational infections in this age group. However, while PCR analysis can reflect an infection in its acute stage, IgM antibodies persist much longer, allowing one to detect an infection for a few months. In addition, the extended storage of the samples, coupled with the fact that those of the patient mothers were exposed to an additional thawing, may have caused a bias in the PCR analysis. It has also been suggested that cellular elements in blood sequester enteroviruses [32] and that whole blood might be better for PCR analysis.

Some earlier reports have shown an association between maternal virus infection during pregnancy and diabetes later in life. Congenital rubella increased the risk of diabetes in the second and third decades of life [13–15], indicating the possibility of an extensive time lag. Other studies have suggested that gestational enterovirus infections may be a precursor of diabetes in young children [21–23]. In one of these studies, the levels of enterovirus antibodies were found to be elevated in the mothers of children who developed type 1 diabetes before the age of three years [21]. In another study tending to corroborate these results, mothers whose children developed type 1 diabetes before age 15 showed an elevated number of enterovirus infections during pregnancy, compared to controls [24]. A case of neonatal diabetes with evidence of maternal enterovirus infection during pregnancy has also been reported [33]. It is known that enterovirus infections show seasonal variation [19]. In an analysis such as ours it is possible that controls matched for time of sampling also have been affected. Their virus antibodies may therefore rather mask a relationship between gestational enterovirus infection and development of type 1 diabetes.

A research project using a larger cohort than those cited above tested for the presence of enterovirus IgM in more than 900 mothers of children who developed type 1 diabetes. Control mothers were carefully matched by the same method employed in our study [34]. No significant difference appeared between case and control groups. However, the samples in that study were taken at the end of the first trimester, thus revealing only infections that occurred during the initial three months of pregnancy. This differs from our study, where samples were taken at delivery, enabling an IgM assay to detect infections arising during the last two trimesters of pregnancy. In addition, the present study included children who were diagnosed with diabetes at an older age than previously investigated. One report including only 16 mothers, most of whom already had type 1 diabetes from the German Multicenter BABY-DIAB study [35], did not support the hypothesis of enterovirus infections during pregnancy causing type 1 diabetes in offspring.

We found an increased risk of developing type 1 diabetes for boys born to IgM-positive mothers. A similar male proclivity towards risk for *β*-cell damage in enterovirus-induced diabetes was found earlier in mice and in prospective studies evaluating the risk effect of postnatal enterovirus infections [36, 37]. Other studies have also shown that boys might be more susceptible to enterovirus infections, possibly due to having a weaker immune system [38]. HLA-DR alleles, which mediate increased risk of type 1 diabetes (DR3 and DR4), have been associated with a stronger humoral response to enterovirus antigens [39] compared to HLA-DR2. In our study, HLA genotypes were not available. This might be a confounding factor, as patient and control subjects ideally should be matched for both sex and HLA type. It is possible that the observed difference between patient and control subjects as the present study noted (also reflected in approximately half of the previous studies cited) indicates a genuine risk effect, particularly in boys, which is later modulated by several postnatal factors.

## 5. CONCLUSIONS

Taken together with previous studies, the present findings suggest that maternal enterovirus infections during pregnancy may affect the risk of type 1 diabetes in offspring. Our data suggest that the risk effect is not pronounced and may be relevant to boys in particular. The risk may also be modulated by factors such as when the infection occurs (early or late in pregnancy), the gender of the child, as well as HLA and other susceptibility genes in the child and the mother. Accordingly, large-scale studies covering the entire period of pregnancy and taking into account children of all ages with type 1 diabetes are warranted.

## Figures and Tables

**Table 1:** Enterovirus findings in serum samples taken at delivery from mothers whose children developed type 1 diabetes in adolescence and young adulthood (Fisher's exact test).

	Index mothers (*n* = 30)		Control mothers (*n* = 90)		*P* value
	Positive *n* (%)	Negative *n* (%)	Positive *n* (%)	Negative *n* (%)	
Enterovirus IgM	9 (30)	21 (70)	14 (16)	76 (84)	0.11
Enterovirus IgM high titers	5 (17)	25 (83)	8 (9)	82 (91)	0.31
Enterovirus RNA	0	30 (100)	4 (4)	86 (96)	0.57

**Table 2:** Odds ratio (OR) and 95% confidence interval (CI) for developing type 1 diabetes (dependent variable in logistic regression) when controlling for mother's age, gender, interaction gender∗ IgM-positivity and IgM-positivity in offspring.

				95.0% CI for	
	B	SE	*P* <	OR	OR
Gender (*M* = 0, *F* = 1)	0.865	0.507	0.088	2.38	0.88–6.42
Gender∗IgM-positive (0/1)	−1.556	1.022	0.128	0.21	0.03–1.56
Mother's age	−0.020	0.044	0.651	0.98	0.90–1.07
IgM-positive (0/1)	1.532	0.682	0.025	4.63	1.22–17.6
Constant	−1.188	1.246	0.340	0.30	

B = regression coefficient of logistic regression elog (OR) SE = standard error for B OR = e^B^.

**Table 3:** Frequency of enterovirus IgM in mothers of offspring developing type 1 diabetes and controls, divided with regard to gender.

Gender of offspring	Maternal enterovirus IgM *n* (%)		Total *n* (%)
	Positive	Negative	
Type 1 diabetes—males	6 (43)	8 (57)	14 (100)
Type 1 diabetes—females	3 (19)	13 (81)	16 (100)
Controls—males	7 (13)	45 (87)	52 (100)
Controls—females	7 (18)	31 (82)	38 (100)
